# Val‐Val‐Tyr‐Pro protects against non‐alcoholic steatohepatitis in mice by modulating the gut microbiota and gut‐liver axis activation

**DOI:** 10.1111/jcmm.16229

**Published:** 2021-01-05

**Authors:** Xinshu Xie, Lang Zhang, Shun Yuan, Huilan Li, Chaojun Zheng, Saisai Xie, Yongbing Sun, Changhua Zhang, Rikang Wang, Yi Jin

**Affiliations:** ^1^ National Pharmaceutical Engineering Center for Solid Preparation in Chinese Herbal Medicine Jiangxi University of Traditional Chinese Medicine Nanchang China; ^2^ Jiangxi Provincial Children's Hospital Nanchang China; ^3^ Guangdong Provincial Key Laboratory of New Drug Design and Evaluation School of Basic Medical Sciences Shenzhen University Health Science Centre Shenzhen China

**Keywords:** anti‐inflammatory, gut microbiota, lipid metabolism, NASH, VVYP

## Abstract

Val‐Val‐Tyr‐Pro (VVYP) peptide is one of the main active components of Globin digest (GD). Our previous studies indicated that VVYP could protect against acetaminophen and carbon tetrachloride‐induced acute liver failure in mice and decrease blood lipid level. However, the effects and underlying mechanisms of VVYP in the treatment of non‐alcoholic steatohepatitis (NASH) have not been discovered. Our present study was designed to investigate the preventive effect of VVYP on NASH and its underlying specific mechanisms. We found that VVYP inhibited the cytotoxicity and lipid accumulation in L‐02 cells that were exposed to a mixture of free fatty acid (FFA). VVYP effectively alleviated the liver injury induced by methionine‐choline‐deficient (MCD) diet, demonstrated by reducing the levels of serum alanine aminotransferase (ALT)/aspartate aminotransferase (AST)/triglycerides (TG)/non‐esterified fatty acids (NEFA) and improving liver histology. VVYP decreased expression levels of lipid synthesis‐related genes and reduced levels of the proinflammation cytokines in the liver of mice fed by MCD diet. Moreover, VVYP inhibited the increased level of LPS and reversed the liver mitochondria dysfunction induced by MCD diet. Meanwhile, VVYP significantly increased the abundance of beneficial bacteria such as *Eubacteriaceae*, *coriobacteriacease, Desulfovibrionaceae, S24‐7* and *Bacteroidia* in high‐fat diet (HFD)‐fed mice, however, VVYP reduced the abundance of *Lactobacillus*. Moreover, VVYP conferred the protective effect of intestinal barrier via promoting the expression of the mucins and tight junction (TJ)‐associated genes and inhibited subsequent liver inflammatory responses. These results indicated that the protective role of VVYP on NASH is mediated by modulating gut microbiota imbalance and related gut‐liver axis activation. VVYP might be a promising drug candidate for NASH.

## 1 INTRODUCTION

Non‐alcoholic fatty liver disease (NAFLD), the most common form of adult chronic liver disease with a prevalence of approximately 25% ~ 30% of the worldwide,^1^ consists of a broad spectrum of disease ranging from simple steatosis to non‐alcoholic steatohepatitis (NASH), causing liver fibrosis, and ultimately cirrhosis with a high risk of hepatocellular carcinoma (HCC).^2^ NAFLD pathogenesis is associated with various types of insults that occur simultaneously and may work synergistically including enhanced accumulation of triglycerides, mitochondrial injury, elevated oxidative stress, autophagy and apoptosis imbalance, increased levels of lipotoxicity and liver inflammation.^3^, ^4^ Effective therapies for treating and preventing NASH are lacking, a single‐targeted drug may not be sufficient to treat NASH which is a metabolic diseases involving multiple factors. Given the complexity of the physiopathology of NASH, a multifunctional drug with two or more targets may provide a better therapeutic effect against NASH than a single‐targeted one.

Stable‐isotope studies showed that de novo lipogenesis (DNL) was increased in patients with NAFLD, which contributed to fat acids accumulation within the liver and the progression of NAFLD.^5^ The most important pathway that adjusts the initiation of fatty acid biosynthesis in the liver involves activation of sterol regulatory element‐binding protein 1 (SREBP‐1c), an important transcription factor involved in hepatic lipid synthesis.^6^ Overexpression of SREBP‐1c in the liver lead to the onset of severe hepatic steatosis because of the increased lipogenesis. The active form of SREBP‐1c promotes fatty acid biosynthesis by activating several downstream lipogenic enzymes, such as fatty acid synthase (FASN) and stearoyl‐CoA desaturase (SCD)‐1.^7^, ^8^ SCD‐1 was responsible for catalysing fatty acid desaturation^9^ and FASN played a crucial role in catalysing the synthesis of palmitate (16:0), which was used for both de novo biosynthesis of ceramide and triglyceride synthesis.^10^ Therefore, the maintenance of normal levels of lipogenesis‐related genes may provide therapeutic benefits in NASH.

Recently, many studies indicated that the progression of NAFLD was associated with the gut‐liver axis.^11^, ^12^ The concept of gut‐liver crosstalk in NAFLD development indicated a connection between increased intestinal epithelial barrier permeability and serum lipopolysaccharide (LPS) level which is a critical driver of hepatic inflammation.^13^, ^14^, ^15^ In general , the composition of the intestinal microbiota reflects the diets, anti‐biotic and the other environment factors of the host,^16^ compelling evidence has demonstrated the gut microbiota played an important role in development of NAFLD to NASH.^17^ Lower gut microbial richness and diversity were observed in NASH patients compared to healthy controls.^18^ Gut microbiota dysbiosis promoted the influx of harmful substances, including LPS, bacterial DNAs and ethanol, into the liver through systemic circulation of portal vein circulation and accelerated the development of NASH.^19^, ^20^ Considerable evidence indicated that chronic inflammation^16^ and intestinal barrier^21^ played critical roles in metabolic diseases induced by gut microbiota disturbance. The combination of the mucus layers and epithelial tight junctions (TJs) formed a highly integrated barrier system that limited luminal contents contact with the host.^22^ Mucins such as the secreted Mucs (Muc‐2, Muc‐3) and membrane associated (Muc‐1) are the major components of the intestinal mucus layer,^23^ which is responsible for maintaining the barrier function of the gut and protecting the epithelium from viruses, pathogenic bacteria and noxious agents present in the gastrointestinal tract.^24^ The tight junction between epithelial cells is comprised of transmembrane proteins (junctional adhesion molecules, claudins, occludin) and scaffold proteins (zonula occludens‐1(ZO‐1), zonula occludens‐2 (ZO‐2), etc) that link the transmembrane proteins to the cytoskeleton, especially the tight junction at the top of the cells plays an important role in the regulation of mucosal permeability.^22^ Recent studies reported that C57BL/6 mice fed by MCD diet^25^ or HFD^26^ impaired intestinal epithelial barrier function by decreasing expression of the TJ proteins in epithelial cells. Dysbiosis of intestinal barrier and ultimately bacterial translocation could trigger profibrogenetic and proinflammatory pathways, finally caused cirrhosis development.^27^ Thus, targeting gut‐liver crosstalk may be an effective approach to mitigate the development of NASH.

Globin digest (GD) is an edible oligopeptide mixture which is hydrolysed of porcine haemoglobin by acid protease.^28^ GD has been used as a specific health food in Japan and it can improve hyperlipidaemia and hyperglycaemia in humans.^29^ Moreover, GD inhibited the increase in serum transaminase activity and showed hepatoprotective effects in liver injury induced by galactosamine (GalN) in Sprague Dawley (SD) rats.^30^ Val‐Val‐Tyr‐Pro (VVYP) is one of the main active components of GD. It could promote the activity of triacylglycerol lipase and reduce blood triglyceride levels,^31^, ^32^ the lipid‐lowering ability of VVYP is 7000 times than that of GD.^28^ In our previous studies, VVYP had obvious protective effect against acetaminophen and carbon tetrachloride‐induced acute liver failure in mice.^33^ However, there is still little known about VVYP for the treatment of NASH. Therefore, the present study is aimed to investigate the protective effects of VVYP on models of NASH induced by MCD and HFD and its mechanisms of these effects.

## 2 MATERIALS AND METHODS

### 2.1 Animals

Forty‐eight male 8‐week‐old C57BL/6 mice were purchased from Hunan SJA Laboratory Animal Co., Ltd. All animals were housed in a specific‐pathogen‐free barrier facility with controlled conditions (19‐23°C, humidity 60%, 12‐h light/dark cycle) and had free access to food and water. After 3 days of adaptive adaptation, the initial body weight was recorded. MCD diet was added to C57BL/6 mice to establish NASH mode for 2 weeks, 28 mice were randomly divided into four groups (n = 7 per group) as follows: (a) a control group (CTL) fed with a normal diet (b) a MCD group fed with a MCD diet (No. 11002900039337, Beijing Keao Xieli Feed Co.,Ltd.); (c) a VVYP 2 group fed with MCD diet and treated with VVYP (2 mg/kg daily by oral gavage); and (d) a VVYP 10 group fed with MCD diet and treated with VVYP (10 mg/kg daily by oral gavage). HFD was used to establish NASH mice model up to 8 week, 20 C57BL/6 mice were divided at random into four groups: CTL group, model(M) group, model +VVYP 10 mg/kg (M‐VVYP), VVYP 10 mg/kg (VVYP). CTL group and VVYP group were fed with normal diet, M and M‐VVYP group were fed with HFD. The body weights were recorded every 2 days. All animal experiments on mice were conducted according to the guidelines approved by the Animal Ethics Committee of Jiangxi University of Traditional Chinese Medicine (approval number JZLLSC 2018‐0053).

### 2.2 Cell culture and treatments

L‐02 cells (Cell Bank, Chinese Academy of Science) were maintained in Roswell Park Memorial Institute (RPMI) 1640 (Solarbio) supplemented with 10% foetal bovine serum (FBS) (Gibco), 100 U/mL of penicillin and 100 μg/ml streptomycin at 37°C with 5% CO_2_. The cell lines were subcultured by trypsinization using 0.25% Trypsin (Solarbio)‐ethylenediaminetetraacetic acid (EDTA). Next, L‐02 cells were seeded at 8000 cells per well in 96‐well plates. Cells were allowed to attach for 24 hours prior to treatments. The FFA mixture (oleate and palmitate, 2:1; Sigma) was prepared with 0.25% defatted bovine serum albumin (BSA) (Sigma). The cells were cultured with different concentrations of FFA mixture and VVYP and cell survival was determined using cell counting Kit (CCK)‐8 (MedChemExpress) according to the manufacturer's protocol. The optic density (OD) value was measured at the detection wavelength of 450 nm. A cell model of NASH was established by exposing L‐02 cells to FFA mixture at a final concentration of 500 μmol/L for 24 hours, and the cell viability of NASH cells cultured with different concentrations of VVYP (3.75, 7.5, 15 μmol/L ) was detected by CCK8 reagent.

### 2.3 Oil red O staining for detecting lipid deposition in L‐02 cells

Cells were processed by oil red O (Solarbio) staining to assess lipid content. The cells were washed three times with phosphate buffered saline (PBS), fixed in 4% paraformaldehyde for 10 minutes, washed twice with ddH_2_O to remove paraformaldehyde. After once wash in 60% isopropanol, the cells were stained with oil red O for 10 minutes at 37°C. Sixty per cent isopropanol were then added to each well and adjusted colour under the microscope. After three washes in ddH_2_O, the cells were synchronized with 60% isopropanol and then dyed with haematoxylin for 30 seconds. Finally, hydrochloric acid alcohol was used to differentiate the background for 3 seconds before microscopic examination. The results were statistically analysed using Image J software.

### 2.4 Animal sacrifice and sample collection

After experimental period, faecal samples were collected from all mice upon defecation and stored at −80°C for further analysis. Mice were fasted for 12 hours, their final body weights were recorded. And then mice were killed. Freshly dissected liver was washed out in ice‐cold physiological saline, and dried with filter paper. The liver was then weighed (in grams), the blood and liver tissues of all groups were collected for the following analysis. All serum samples were stored in a −80°C freezer. Liver and small intestine samples were snap‐frozen in liquid nitrogen or kept in a −80°C freezer for further procedures.

### 2.5 Biochemical analysis

Blood was collected at the end of study from each experimental animals and allowed to stand for 2 hours to clot. And then the blood samples were centrifuged (4500 rpm, 20 minutes) for serum separation. The biochemical indicators of mice in each group were measured using an auto‐analyser (Hitachi). We determined ALT, AST, total cholesterol (CHOI), TG, NEFA.

### 2.6 Histological studies

#### 2.6.1 Haematoxylin and eosin staining for liver

**Haematoxylin and eosin** staining was performed to detect liver injury and fibrosis. Fresh liver samples were fixed in 4% neutral‐buffered formalin for 72 hours and then processed for sectioning and staining according to standard histological methods. The liver tissue was embedded in paraffin wax and cut into 4 μm slices with Leica microtome (LEICA RM2016). Paraffin was removed and sections were stained with haematoxylin‐eosin dye. The histopathological changes of liver were evaluated under light microscope (LEICA DM 1000).

#### 2.6.2 Oil red O staining for liver

Liver sections were stained with oil red O and haematoxylin to observe lipid droplets. Frozen Liver samples were embedded in optimal cutting temperature (OCT) compound and stored at −20°C. And the samples were then sliced into 8 μm sections with Leica cryostat (LEICA CM1850). The dyeing steps are as follows: (a) Washed by PBS (pH 7.2) for 3 times, 5 minutes per time, (b) 60% isopropanol for 2 minutes, (c) oil red O in 60% isopropanol at 37°C for 5 minutes, (d) 60% isopropanol for 3 minutes, (e) washed in ddH_2_O, (f) haematoxylin for 2 minutes, (g) hydrochloric acid alcohol for 3 seconds. Finally, the slices were sealed by cover glass and observed using a microscope (LEICA DM 1000). The lipid accumulation was statistically analysed using Image J software.

#### 2.6.3 Immunofluorescence staining

Frozen small intestine tissues were embedded in paraffin wax and sectioned at 5 μm with Leica microtome (LEICA RM2016). For immunostaining, sections were incubated with rabbit polyclonal ZO‐1 antibody (1:100, Proteintech) at 4°C overnight and treated with the Goat Anti‐Rabbit IgG H&L(FITC) (1:400, Abcam) as secondary antibody. Then the sections were incubated with DAPI (1:1000) for 3 minutes and imaged using a fluorescent microscope (LEICA DMI300B). Visual fields were randomly selected and analysed with Image J software.

#### 2.6.4 Transmission electron microscopy (TEM) analysis of liver tissue

The liver specimens were fixed in 2.5% glutaraldehyde overnight at 4°C for 24 hours, washed three times in 0.1 mol/L phosphate buffer (pH 7.4) and then post‐fixed in 1% osmium acid solution for 2 hours. Followed by secondary fixation, the specimens were washed briefly as mentioned above. Graded ethanol series dehydration and embedded in epoxy resin. Ultrathin sections (60‐80 nm) were then cut, ultramicrostructure related to lipid droplets and mitochondrial morphology was examined with a transmission electron microscope (Hitachi, HT7700‐SS).

### 2.7 RNA extraction and real‐time PCR analysis

The total RNA was extracted from pulverized frozen liver and small intestinal tissues using Trizol (CWBIO) according to the manufacturer's protocols. Then total RNA purity and content were measured by a spectrophotometer, total RNA (1 μg) from liver tissues and small intestine samples was reverse transcribed to cDNA using a RevertAid First Strand cDNA Synthesis Kit (Thermo Fisher Scientifi, USA). The mRNA expression levels were assessed by qRT‐PCR using the SYBY Green PCR Master Mix (Thermo Fisher Scientific) and the ABI 7500 Real‐time PCR system (Applied Biosystems). The relative expression of each gene was normalized to glyceraldehyde‐3‐phosphate dehydrogenase (GADPH). Primer sequences used are listed in Table 1.

**TABLE 1:** Sequence of primers for quantitative real‐time PCR

Gene name	Forward primer sequence (5′‐3′)	Reverse primer sequence (5′‐3′)
Mouse		
GADPH	GGAGAAACCTGCCAAGTATGATGAC	GAGACAACCTGGTCCTCAGTGTA
FASN	ATTCGTGATGGAGTCGTGAAG	GGTCTTGGAGATGGCAGAAAT
SCD1	GGTCTTGGAGATGGCAGAAAT	GGTCTTGGAGATGGCAGAAAT
ZO‐1	CGGAACTATGACCATCGCCTAC	CTTCGGGATGTTGTCTGGAGTC
Claudin‐1	AGCTGTGCATGGCCTCTTGT	CCAATGTCAATGGAACACCCT
Occludin‐1	CAGCCTCGGTACAGCAGCAAT	ATAGTGGTCAGGGTCCGTCCTC
Muc‐1	AATGGCTCCTCGGTGCTACCTA	TGACTTGGCACTGAAGGCTGAG
Muc‐2	TGCTGACGAGTGGTTGGTGAATG	GATGAGGTGGCAGACAGGAGACA

### 2.8 Western blot analysis

Liver samples were randomly selected from each group, and total proteins were extracted from approximately 50 mg of liver with 500 μl radioimmunoprecipitation assay (RIPA) lysis buffer (Solarbio, USA) containing phosphatase and protease inhibitors. Next, the mixture was centrifuged at 12 000 *g* for 5 minutes at 4°C and the supernatant was extracted. The protein content was estimated using a BCA Protein Assay Kit (Cwbiotech). Equal amount of proteins were separated on 7.5% or 10% sodium dodecyl sulphate polyacrylamide gel electrophoresis (SDS‐PAGE) gels and sequentially transferred onto polyvinylidene fluoride (PVDF) membranes (Millipore). After blocked with 5% skimmed milk powder for 2 hours, the membranes were incubated with the specific antibodies for FASN (1:800; Abcam), SCD‐1 (1:1000; Cell Signaling Technology), SREBP‐1c (1:800; Affinity Biosciences) and β‐actin(1:1000; Abcam) at 4°C overnight. The next day, the membranes were washed and incubated with secondary antibodies for 1 hours at room temperature, goat anti‐rabbit (N10429) IgG‐HRP and the goat antimouse IgG‐HRP (N10326) secondary antibodies were purchased from TransGen Biotech. The membranes was placed in an electrochemiluminescence (ECL) Western blotting detection system (Bio‐Rad), ECL detection reagent (Cwbiotech) was added, exposure and visualization were performed, and grey values of each band were analysed using Image J software.

### 2.9 Enzyme‐linked immunosorbent assay (ELISA)

Tumour necrosis factor (TNF)‐α, interleukin (IL)‐6 and LPS levels in liver tissues were quantified using commercial ELISA kits (WESTANG BIO‐TECH), based on the manufacturer's instructions.

### 2.10 16S rDNA gene sequencing and analysis

Extraction of DNA from different faecal samples were performed using the EZNA^®^ soil DNA Kit (Omega Bio‐tek) according to the manufacturer's protocols. The final DNA purification and concentration were detected by NanoDrop 2000 UV‐vis spectrophotometer (Thermo Scientific), and DNA quality was determined by 1% agarose gel electrophoresis. The V3‐V4 variable regions of the bacteria 16S rRNA gene were then amplified with primers 338F (5′‐ACTCCTACGGGAGGCAGCAG‐3′) and 806R (5′‐GGACTACHVGGGTWTCTAAT‐3′) by thermocycler PCR system (GeneAmp 9700, ABI). Purified amplicons were paired‐end sequenced on an Illumina MiSeq platform (Illumina) according to manufacturer's procedures. Sequence analysis was performed using the QIIME2 feature‐table plugin. The operational taxonomic units (OTUs) and the representative read for each OTU were selected by UCLUST method. Identification of the bacteria with different abundance in different samples and groups was performed using ANCOM, ANOVA, Kruskal‐Wallis, DEseq2 and linear discriminant analysis effect size (LEfSe) methods.^34^, ^35^ The core‐diversity plugin within QIIME2 was employed to calculate diversity metrics. α‐diversity indices, such as observed species, Shannon index and Faith index were calculated to assess the bacterial diversity within an individual sample. β‐diversity analyses, including unweighted UniFrac, weighted UniFrac and Bray Curtis were performed to examine the structural variation of microbial communities among samples and then visualized via principal coordinate analysis (PCoA) and non‐metric multi‐dimensional scaling (NMDS).^36^

### 2.11 Statistical analysis

Statistical analysis was performed using GraphPad 8.0 statistical package (GraphPad Software) and the graphs were also generated with Prism. Results were expressed as the means ± SEM. Two‐tailed Student's *t*‐test was performed to measure the difference between two sets of data. The variance of three or more groups was determined by one‐way ANOVA and the Bonferroni post‐hoc analysis. Kruskal‐Wallis and Wilcoxon tests were used to perform LEfSe analysis associated with relative abundance of gut microbiota and the threshold on linear discriminant analysis (LDA) score was high than 3. Others were displayed using QIIME1 and R packages (V.2.15.3). For all statistical tests, *P* values < .05 were considered to indicate statistically significant.

## 3 RESULTS

### 3.1 VVYP improved cell viability and steatosis of L‐02 cells induced by FFA

**FIGURE 1:**
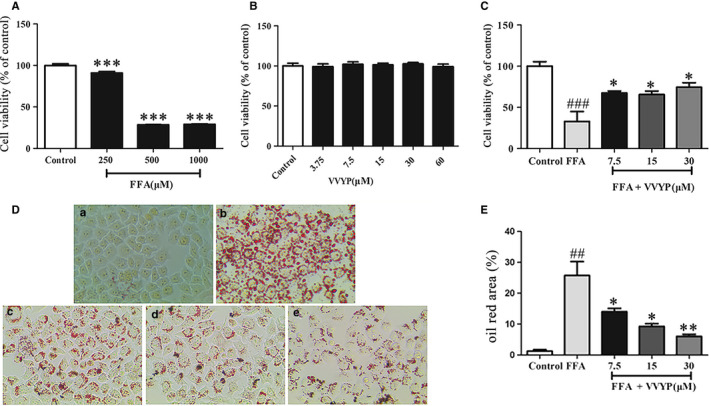
Effect of VVYP on cytotoxicity and lipid accumulation in L‐02 cells. A, NASH cell model was established by exposing L‐02 cells to FFA, L‐02 cells were exposed to the different concentrations of FFA (250 μmol/L, 500 μmol/L and 1000 μmol/L) for 24 h. The cell viability was measured by CCK‐8. B, Cells were cultured with different concentrations of VVYP (3.75, 7.5, 15, 30 and 60 μmol/L) for 24 h. The cell viability was assessed with CCK‐8. C, NASH cells were cultured in different concentrations of VVYP (3.75, 7.5 and 15 μmol/L) for 24 h and viability of cells was assessed with CCK‐8. D, Lipid accumulation were stained in NASH cells cultured with or without VVYP with oil red O. The magnifications were 200×. C(a) Normal control group; C(b) FFA 250 μmol/L (model group); C(c) VVYP 7.5 μmol/L (VVYP‐D group); C(d) VVYP 15 μmol/L(VVYP‐M group); C(e) VVYP 30 μmol/L (VVYP‐H group). E, Statistical analysis of oil red area data in (D). All results were repeated three times, representative data were expressed by mean ± SEM. ***P < .01 vs control group, ##*P* < .01 vs control group, ###*P* < .001 vs control group, *P < .05 vs FFA group (model group), **P < .01 vs FFA group (model group)

Previous studies have reported that treatment of L‐02 cells with FFA served as an useful in vitro NASH model. FFA could cause hepatic injury in vitro, with obviously inducing apoptosis of L‐02 cells. To evaluate the optimal concentrations of FFA and VVYP, L‐02 cells were treated with different concentrations of FFA and VVYP for 24 hours and the cytotoxicity of FFA and VVYP was measured by CCK‐8. As shown in Figure 1A, cell viability was obviously decreased when the FFA concentration was at 500 μmol/L and 1000 μmol/L respectively, while 500 μmol/L FFA was the lowest dosage that caused the cell viability more than 10%. Hence, we used L‐02 cells treated with 500 μmol/L FFA as a NASH model in vitro. As shown in Figure 1B,C, no apparent cytotoxic effect of VVYP was observed when L‐02 cells were cultured with VVYP from 3.75 to 60 μmol/L, and the viability of L‐02 cells induced by FFA was remarkably improved when they were treated with different concentrations of VVYP for 24 hours.

In order to investigate the effect of VVYP on lipid accumulation, after the FFA precultured, L‐02 cells were first exposed to FFA and then treated with various concentrations of VVYP, the lipid deposition was assessed by oil red O staining. Compared with control group, FFA significantly increased number of red lipid droplets, while VVYP dose‐dependently reduced cellular lipid accumulation induced by FFA in L‐02 cells (Figure 1D,E). These results suggested that VVYP could diminish steatosis induced by FFA in L‐02 cells.

### 3.2 Protective effect of VVYP on MCD diet‐induced NASH mice

Mice fed by the MCD diet for 2 weeks developed liver injury and accumulated hepatic lipids. As shown in the Table 2, the levels of AST, ALT, TG and NEFA in the MCD diet group were significantly higher than those of the normal diet group, but VVYP reversed these effects induced by MCD diet. The levels of ALT, AST, TG and NEFA in high dose of VVYP group (10 mg/kg) showed a superior therapeutic effect in comparison with Bicyclol group.

**TABLE 2:** Effects of VVYP on serum biochemical indexes in mice

	ALT (U/mL)	AST (U/mL)	CHOI (mg/dL)	TG (mg/dL)	NEFA (mmol/L)
Control	29 ± 5.82	92 ± 13.62	107 ± 27.64	19 ± 9.00	1.04 ± 0.20
MCD	136 ± 42.42###	238 ± 60.95###	127 ± 1.47	32 ± 7.86#	1.51 ± 0.29##
Bicyclol	47 ± 10.50***	111 ± 15.66***	124 ± 8.61	31 ± 8.08	1.35 ± 0.30
VVYP 2 mg/kg	45 ± 13.31***	99 ± 2.12**	140 ± 13.74	30 ± 4.67	1.53 ± 0.26
VVYP 10 mg/kg	43 ± 8.73***	97 ± 2.79***	151 ± 11.32	19 ± 2.71**	1.19 ± 0.12*

Note

Data are shown as means ± standard deviations (n = 7).

^#^ *P* < .05, ^##^ *P* < .01, ^###^ *P* < .001 vs Control; ^*^ *P* < .05, ^**^ *P* < .01, ^***^ *P* < .001 vs MCD.

**FIGURE 2:**
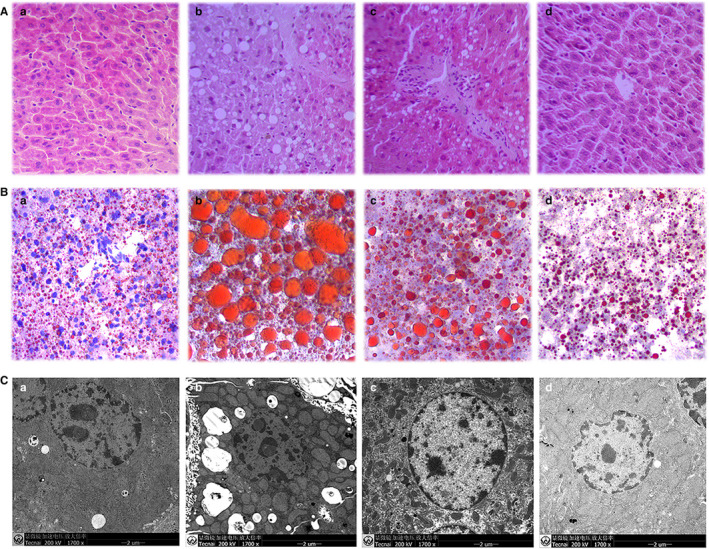
VVYP ameliorated histological characteristics of NASH induced by MCD diet. A, Histopathologic analysis (inflammatory cells and accumulation of fat) of liver tissues in each group was detected by H&E staining (magnification 200 ×). A(a) Control group; A(b) MCD group; A(c)VVYP 2 mg/kg (VVYP‐D group); A(d) VVYP 10 mg/kg (VVYP‐H group). B, Effect of VVYP on lipid accumulation was identified by oil red O (magnification 200 ×). B(a) Control group; B(b) MCD group; B(c)VVYP 2 mg/kg (VVYP‐D group); B(d) VVYP 10 mg/kg (VVYP‐H group). C, Ultramicrostuctures changes of mitochondria and lipid droplets in livers were observed by transmission electron microscopy. C(a) Control group; C(b) MCD group; C(c)VVYP 2 mg/kg (VVYP‐D group); C(d) VVYP 10 mg/kg (VVYP‐H group). Scale bar: 2 μm

HE staining was applied to observe liver tissue morphology and inflammatory cell infiltration, and oil red O staining was used to visualize lipid droplets in liver cells. As shown in the Figure 2A,B, MCD diet‐fed mice were prominently appeared to be steatosis with accumulated micro‐ and macro‐vesicular fatty droplets, thus leading to hepatocyte ballooning as well as increased inflammatory cell infiltration. In contrast, livers from MCD diet‐fed mice treated with VVYP had more regular structure and morphology of hepatic cells compared with MCD diet group. Interestingly, lipid macro‐ and micro‐vesicles were almost absent in the liver of the mice when they were treated with high dose of VVYP group (10mg/kg). We further confirmed these results using transmission electron microscopy (TEM). The normal diet‐fed mice showed normal ultrastructure and had no apparent changes (Figure 2C), while the morphological structure of liver mitochondria was damaged in the MCD diet group, VVYP reversed the effects of MCD diet by significantly preventing liver lipids infiltration, inhibiting mitochondrial vacuolation and overall maintaining the regular liver ultrastructure and histology.

### 3.3 VVYP neutralized MCD diet‐induced up‐regulation of FASN, SCD1 and SREBP‐1c in mice

**FIGURE 3:**
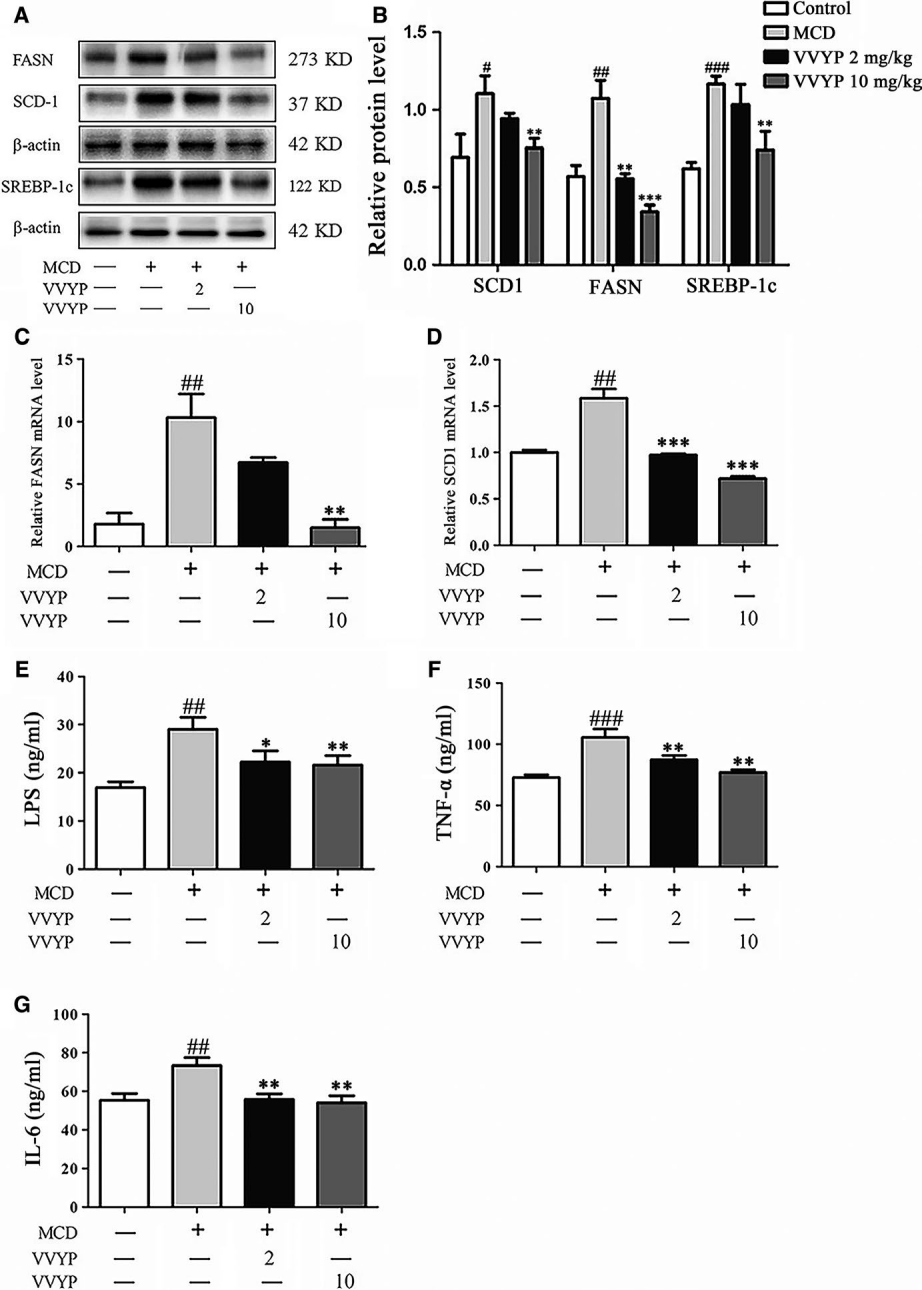
Effect of VVYP on inhibiting excessive lipogenesis and expressions of proinflammatory cytokines. A, Evaluation of SCD‐1, FASN and SREBP‐1c protein content in liver tissues. B, Bar chart representing the relative protein expression level. C and D, Evaluation of FASN and SCD‐1 mRNA levels in liver tissues among the different experimental groups. E‐G, Levels of LPS, TNF‐α and IL‐6 in liver tissues among the different experimental groups were detected with ELISA. Each bar represents the mean ± SEM. #*P* < .05, ##*P* < .01, ###*P* < .001 vs Control; *P < .05,**P < .01,***P < .001 vs MCD

In order to investigate the underlying mechanism of the protective effect of VVYP on NASH, three critical lipogenesis genes involved in DNL were detected. Western blot analysis revealed that the protein expressions of FASN, SCD1 and SREBP‐1c were up‐regulated in MCD diet‐fed mice compared with the normal diet‐fed mice, while VVYP significantly decreased the levels of FASN, SCD1 and SREBP‐1c in MCD diet‐fed mice in a dose‐dependent manner (Figure 3A,B). Consistently, VVYP inhibited the mRNA levels of FASN and SCD‐1 in the livers of MCD diet‐fed mice (Figure 3C,D).

### 3.4 VVYP attenuated inflammatory responses in MCD diet‐induced NASH mice

Inflammation plays an important role in the progression of NASH, and levels of proinflammatory cytokines reflect the strength of the immune response. Endotoxin or LPS, a cell‐wall component of bacteria sensed by toll‐like receptor 4 (TLR4), has been implicated as a potent second hit and results in inflammasome activation as well as progressive inflammatory injury. We measured the levels of cytokines and LPS by ELISA. As exhibited in the Figure 3E‐G, the levels of LPS, TNF‐α and IL‐6 were significantly increased in the livers of MCD diet‐fed mice in comparison with normal control. However, VVYP administration obviously abolished these elevations induced by MCD diet. These data indicated that VVYP decreased inflammatory responses in the mice fed by MCD diet.

### 3.5 VVYP preserved intestinal barrier function in the small intestine of MCD diet‐fed mice

**FIGURE 4:**
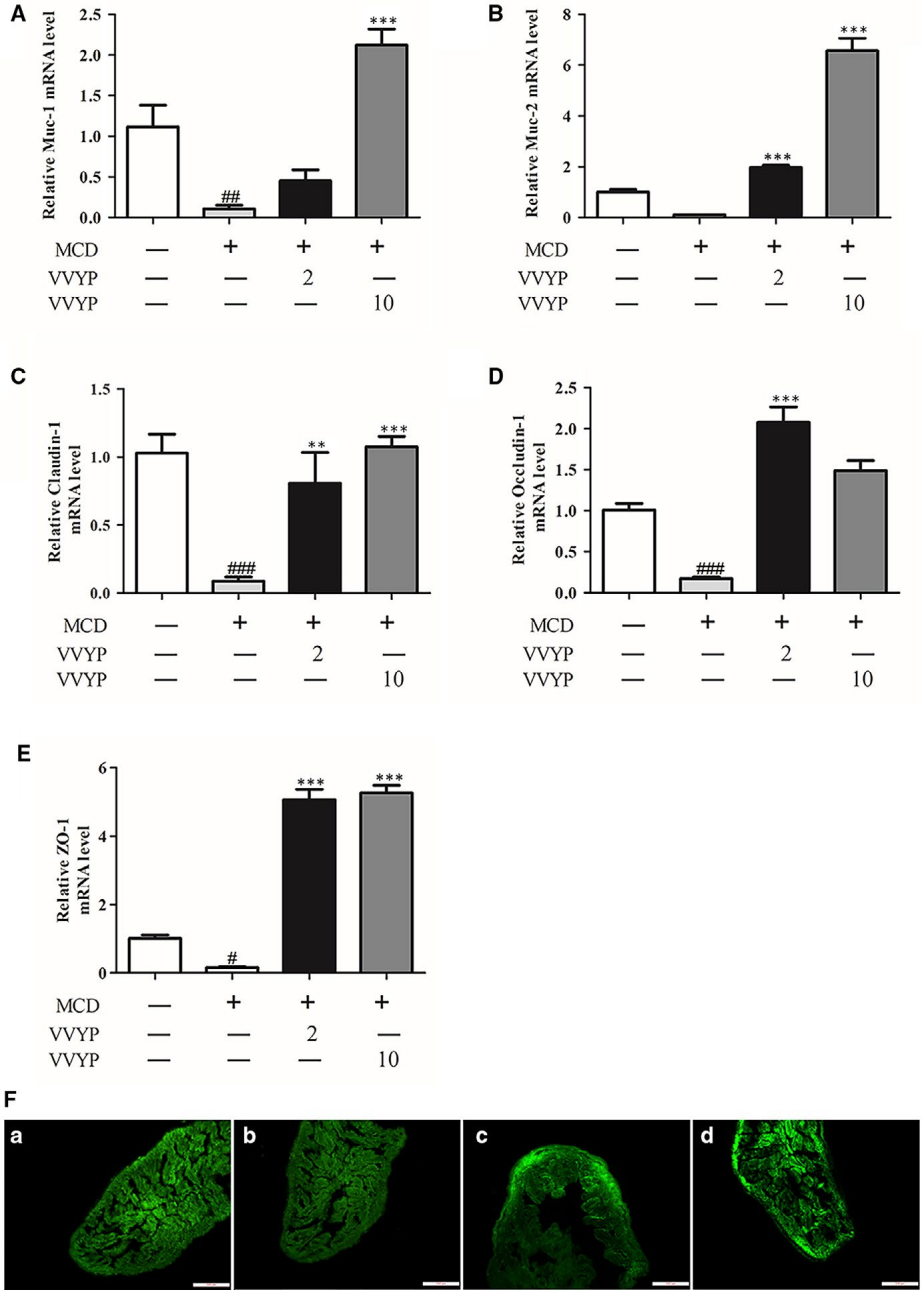
VVYP improved intestinal barrier function in the small intestine. A‐E, Relative mRNA levels of the mucins (Muc‐1, Muc‐2) and TJ (tight junction)‐associated genes (claudin‐1, occluding‐1, ZO‐1). F, Evaluation of representative small intestine histology by ZO‐1 immunofluorescence (scale bar, 200 μm). F(a) Control group; F(b)MCD group; F(c) VVYP 2 mg/kg (VVYP‐D group); F(d) VVYP 10 mg/kg (VVYP‐H group). Each bar represents the mean ± SEM. #*P* < .05, ##*P* < .01, ###*P* < .001 vs Control; **P < .01, ***P < .001 vs MCD

Numerous studies have reported that intestinal barrier dysfunction played essential roles in the progression of NASH. To verify the potential effects of VVYP on intestinal barrier function, the mRNA levels of the mucins (Muc‐1, Muc‐2) and TJ‐associated genes (ZO‐1, claudin‐1, occluding‐1) were measured in small intestine tissues by RT‐PCR. As shown in Figure 4A‐E, the gene expressions of Muc‐1, Muc‐2, ZO‐1, claudin‐1 and occluding‐1 were observably suppressed by MCD diet in comparison with the normal control, while VVYP was shown to significantly increase the expression of these genes. In addition, immunofluorescence assay was further confirmed the expression of ZO‐1 in intestinal epithelial tissue. As shown in Figure 4F, compared with the normal diet group, the disruption level of ZO‐1 was decreased in the damaged intestinal sections of the MCD diet group, which were dramatically recovered by VVYP administration. Hence, VVYP might reverse intestinal mucosal barrier damage triggered by MCD diet via enhancing the expression of the mucins and tight junctions makers.

### 3.6 VVYP restored the diversity, richness of the gut microbiota in HFD‐fed mice

**FIGURE 5:**
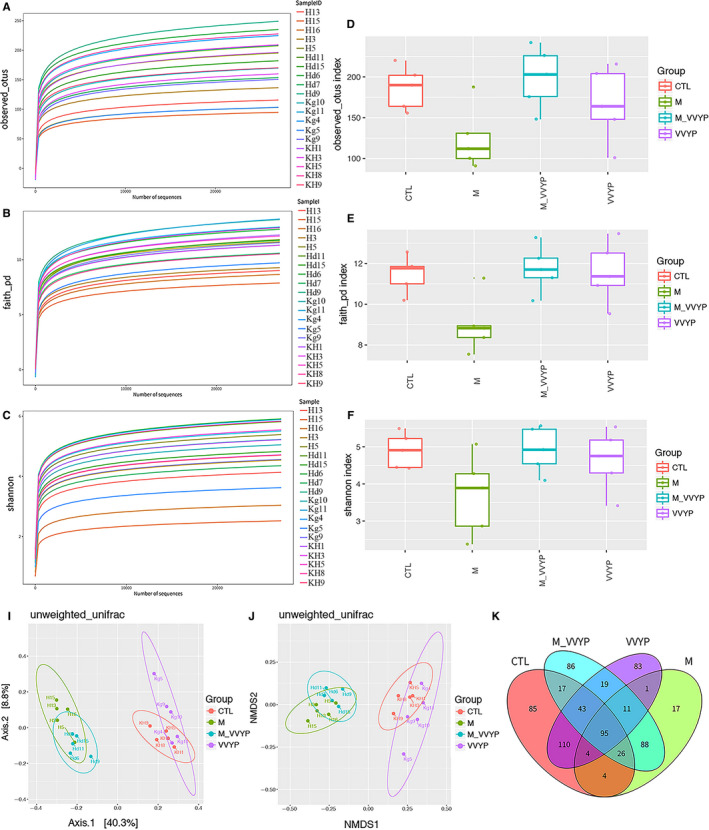
Influences of VVYP on the diversity, richness of the gut microbiota. C57 mice were divided into four groups (CTL, M, M‐VVYP, VVYP). A‐C, Refraction curve of observed index, Faith index and Shannon index. D, observed index. E, Faith index. F, Shannon index. I, principle component analysis (PCoA). J, non‐metric multidimensional scaling (NMDS) analysis. K, Venn diagram was used to show the common and unique operational taxonomic units between four groups

Since intestinal microbial dysregulation is another factor in NASH and liver‐gut axis plays a key role in various diseases such as obesity, NALFD and NASH, we determined the structural changes of gut microbiota by 16S rDNA sequencing of mice faecal samples and we observed distinct alterations in the microbial ecology in CTL, M, M‐VVYP and VVYP groups. As shown in Figure 5A‐C, the rarefaction curves reached a plateau with the current sequencing, which reflected that the majority of microbial diversity had been captured in all samples. The microbiota α‐diversity metrics were reduced in HFD‐fed mice, such as the observed species, Faith index and Shannon index (Figure 5D‐F), which indicated that the HFD‐fed mice decreased the gut microbiota diversity and richness. However, VVYP increased the diversity indexes of OTU in HFD‐fed mice, and the diversity indexes in VVYP alone group showed no obvious change. In addition, as shown in Figure 5I,J, the HFD diet also obviously altered the β‐diversity and the overall composition of the gut microbiota of the mice. PCoA (Figure 5I) and NMDS (Figure 5J) analysis from the abundance of OTUs showed that the gut microbial community structure segregated differently between control (CTL) and HFD (M) groups, while the two clusters from M group and M‐VVYP group were not entirely separated. The Venn diagram was used to demonstrate the common and unique OTUs, thus intuitively exhibiting sample overlap and similarity. Interestingly, the exclusive OTUs of the M group was the lowest, indicating that HFD destroyed the diversity of gut microbiota, and there was a remarkable difference in the structure of the gut microbiota between HFD‐fed mice and normal diet‐fed mice (Figure 5K). Moreover, VVYP group, M group and M‐VVYP group shared 252, 129 and 181 OTUs with CTL group respectively, indicating that the VVYP could restore the disordered gut microbiota induced by HFD‐fed mice.

**FIGURE 6:**
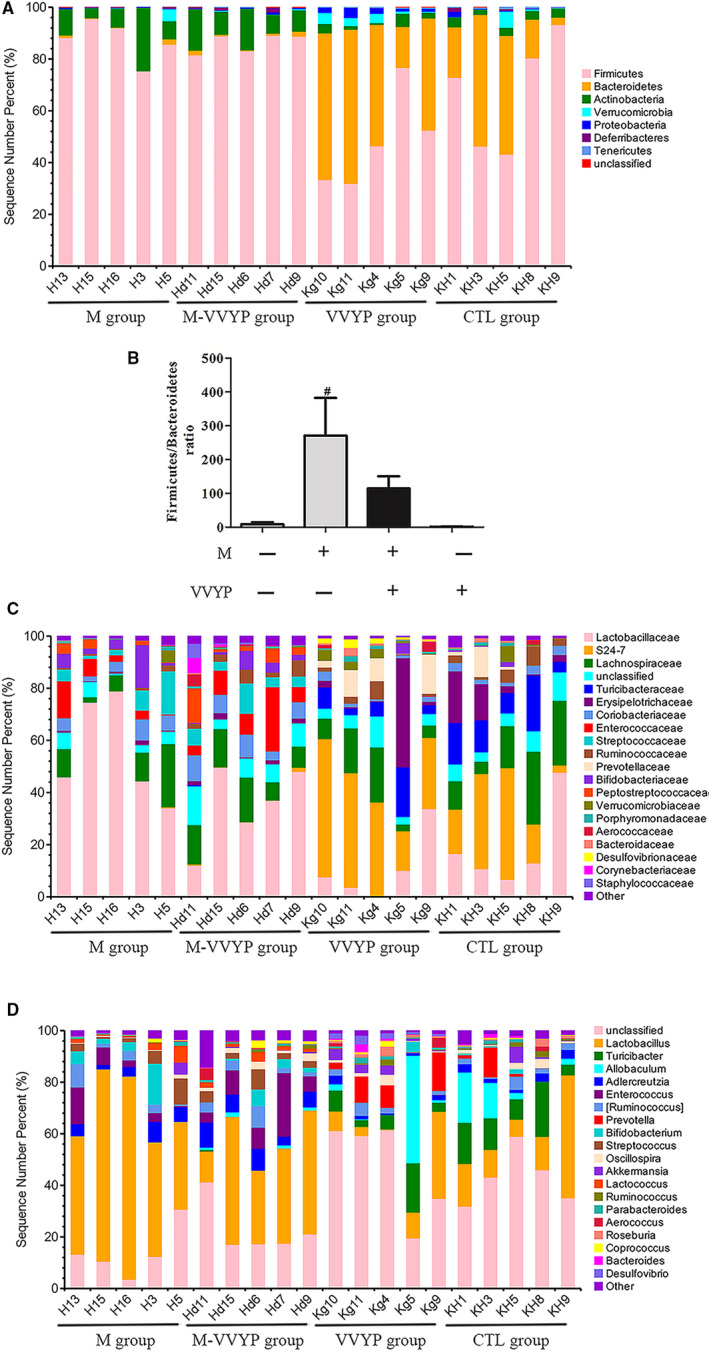
Effects of VVYP on the composition of the gut microbiota in HFD‐fed mice. Hierarchical cluster analysis. A, C and D, Bacterial composition of the different communities at the phylum level (A), family level(C) and genus level(D). B, Bar chart representing the FB ratio. #*P* < .05 vs CTL

Statistics of the OTUs revealed the relative abundance of the gut microbiota at the classification of phylum, family and genus. The results suggested that gut microbial composition was different in the four groups. Most abundant taxa at the phylum, family and genus levels were plotted as stacked bar in Figure 6A,C,D. In all samples, the two most dominant phyla were *Firmicutes* and *Bacteroidetes*. The *Firmicutes‐*to*‐Bacteroidetes* (FB) ratio was considered as a biomarker of intestinal disorder. The *Bacteroidetes* were decreased while *Firmicutes* were increased in HFD‐fed mice, which were accompanied by a higher FB ratio. However, the *Bacteroidetes* was increased and *Firmicutes* was decreased after 8 weeks of VVYP intervention (Figure 6B), moreover, the FB ratio was decreased. At the genus level, the *Lactobacillus* and *Streptococcus* were significantly increased in the M group compared with the CTL group. However, VVYP administration significantly reduced the *Lactobacillus* in the M group.

**FIGURE 7:**
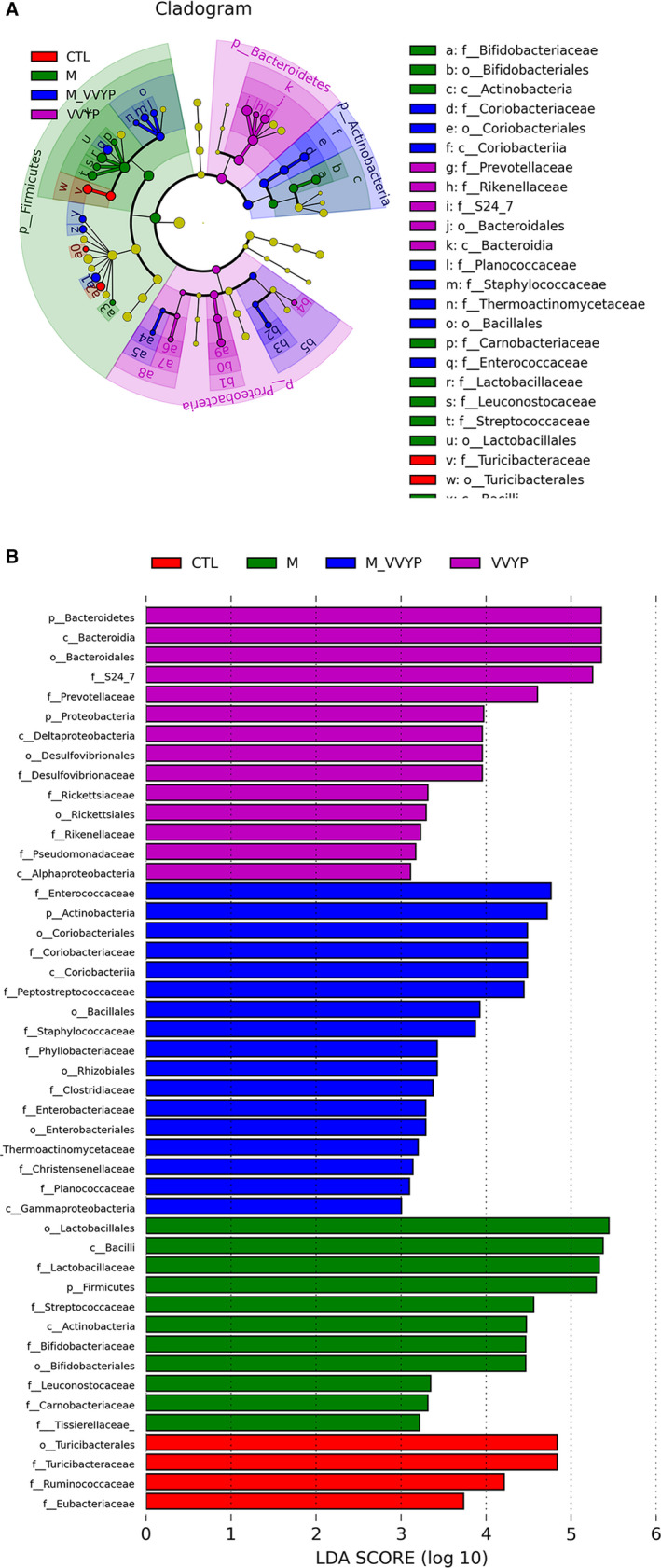
A, LEfSe comparison of gut microbiota among four experimental groups. B, LDA with an LDA score >3 of four experimental groups

The differences of abundance among the CTL group, M group, M‐VVYP group and VVYP group were detected by LEfSe analysis (LDA score > 3). As shown in Figure 7A,B, at family level, *Turicibacteriaceae*, *Ruminococcaceae* and *Eubacteriaceae* were abundant in normal diet‐fed mice, which suggested that these bacteria might exert potential protective effects on NASH induced by HFD. VVYP alone reduced the levels of *Firmicutes* while increased the levels of *Desulfovibrionaceae* and *Bacteroidetes*, in which *Bacteroidia* was the dominant strain at class level, and VVYP also caused an increase in *S24‐7* and *Prevotellaceace* at family level. However, *Firmicutes* was increased in HFD‐fed mice, and HFD caused an increase in *Bacilli* at class level and *Lactobacillaceae* at family level. Feeding NASH mice with VVYP increased *Eubacteriaceae* and *coriobacteriacease* at family level and *Actinobacteria* at phylum level.

## 4 DISCUSSION

NASH is a more severe form of NAFLD, it encompasses a range of distinct pathological features in the liver, including hepatocellular ballooning, hepatocyte injury, liver inflammation, steatosis and fibrosis and can further progress to liver cirrhosis and HCC.^37^, ^38^, ^39^ Our previous study revealed that VVYP could reduce serum AST and ALT, improve the pathological state of liver tissue, and protect against liver injury caused by carbon tetrachloride or paracetamol in mice.^33^ Elevated ALT level is a common marker of progressive NAFLD or NASH and has been correlated with insulin resistance and severity of hepatic steatosis.^40^ In this studies, we found VVYP ameliorated MCD diet‐induced liver injuries via reducing ALT/AST/TG/NEFA levels, promoting lipid deposition, regulating expressions of liver lipogenesis‐related proteins and anti‐inflammatory actions including inhibition of the levels of LPS, TNF‐α and IL‐6 in liver tissue, VVYP treatment could also preserve intestinal barrier function by up‐regulating expression levels of Muc‐1, Muc‐2, Claudin‐1, ZO‐1 and Occludin‐1 and improving the diversity of gut microbiota. Noteworthily, the abundance of probiotics (*Eubacteriaceae*, *coriobacteriacease, Desulfovibrionaceae, S24‐7* and *Bacteroidia)* was enhanced and the abundance of *Lactobacillus* was reduced following VVYP treatment.

Mice fed by MCD diet and HFD have been broadly applied in NASH‐related research.^41^, ^42^ Multiple mechanisms have been proposed in the MCD model, which closely replicated human NASH histological phenotype within a relatively short period.^43^ Methionine deficiency initially resulted in oxidative stress and alterations in adipokines and cytokines, which were considered to drive the development of inflammation in animals, whereas choline deficiency contributed more to the phenotype of hepatic steatosis.^44^, ^45^ Bicyclol is a widely used drug for clinical treatment of various liver injuries, and its hepatoprotective efficacy has been identified in previous researches.^46^, ^47^, ^48^ Therefore, we used bicyclol as a positive control drug in our studies. MCD‐diet feeding caused severe liver damage as demonstrated by the elevated serum level of liver enzymes, especially ALT, which has been designated as a requisite in clinical diagnosis of NASH.^49^, ^50^ In this study, VVYP markedly decreased MCD‐induced elevations of serum AST and ALT, and VVYP showed a superior therapeutic effect in comparison with bicyclol, in addition, high dose of VVYP significantly reduced the levels of TG and NEFA compared with MCD group. The formation and accumulation of lipid droplets ( micro‐ or macro‐vesicles ) and triglycerides in the liver cells has been considered to be the pathological hallmark of NAFLD and has been well accepted to predict stage of the development of fatty liver disease.^51^, ^52^ In the present study, HE staining and oil red O staining experiments exhibited that VVYP significantly ameliorated histological changes and lipid accumulation in mice treated with MCD diet. Furthermore, VVYP significantly reduced lipid droplet formation in a dose‐dependent manner in L‐02 cells which were exposed with FFA. As well known that L‐02 cells treated with FFA are a NASH mode *in vitro*.^53^, ^54^ Mitochondria played a pivotal role in cellular oxidative stress and lipid metabolism, and liver mitochondria dysfunction was supposed to be one of the initial events during the progression of NAFLD.^55^ Inhibition of mitochondrial fission has been proved to block hepatic steatosis and develop to NAFLD.^56^ In our study, TEM results showed that MCD diet caused vacuolated and swollen mitochondria with increased fragmentation in the liver of the mice, however, VVYP reversed these changes in MCD diet‐fed mice and maintained the regular mitochondria ultrastructure and function of the liver cell. Thus, these results confirmed that VVYP improved the lipid metabolism and preserved normal liver ultrastructure and histology.

DNL is a major contributor to the pathogenesis of NAFLD. Previous studies showed that SREBP‐1c^57^ and SCD‐1^58^ levels were up‐regulated in the fatty livers of NAFLD patients, and higher FASN levels were confirmed in human liver samples and the murine model of hepatic steatosis.^59^ In our study, both the mRNA and protein levels of SCD‐1 and FASN were dose‐dependently reduced in VVYP group compared with MCD group. High protein levels of SREBP‐1c induced by MCD diet were significantly reversed by high‐dose VVYP. These data revealed that VVYP could inhibit DNL and reduce hepatic lipid accumulation.

The gut‐liver axis, a bidirectional interplay between intestinal and hepatic diseases, was recently under intense investigation as a critical factor in NAFLD progression.^11^, ^12^ It is well known that the abundance of gut microbiota plays a crucial role in maintaining the stability and efficiency of dynamic balance of micro‐ecosystem.^60^ Targeting the intestinal microbiota has been shown to be beneficial for the therapy of NAFLD.^3^, ^61^ From the analysis of observed species, Shannon index and Faith index, our results indicated that the α‐diversity was lower during the development of NASH induced by HFD, while VVYP treatment up‐regulated these diversity indexes. The Venn diagram intuitively exhibited that VVYP group shared the most OTUs with the control group. Furthermore, the analysis of unweighted UniFrac NMDS and PCoA reflected an obvious clustering of the bacterial community in model group (M, fed by HFD) compared with control group. These results confirmed that HFD changed microbial communities during the development of NASH. The Bacteroidetes was decreased in HFD diet‐fed mice, whereas the *Firmicutes* was increased, leading to a marked higher FB ratio. By contrast, we discovered treatment with VVYP reversed these changes. It has been reported that the FB ratio was increased and there was a distinctly lower proportion of *Bacteroidetes* in mice fed by HFD.^62^, ^63^, ^64^ It should be considered that VVYP improved the gut microbiota composition in mice with NASH. An increase in *Lactobacillus* and *Streptococcus* has been reported in NAFLD patients in comparison with healthy controls.^65^, ^66^ Consistent with previous findings, we found that the *Lactobacillus* and *Streptococcus* were increased in the HFD‐fed mice while VVYP could significantly reduce the *Lactobacillus*. There are more than 180 species of *Lactobacillus*, which have important immune function and various metabolic activity. Indeed, *Lactobacilli* produce lactic acid through the fermentation of dietary ethanol, carbohydrates and acetate ^67^ which is related to liver injury^68^ and higher fibrosis scores^69^ in patients with NASH. In particular, several *Lactobacillus* species produce ethanol^70^, ^71^, ^72^ and oxidize ethanol to acetaldehyde,^73^ which damage the intestinal barrier and increase intestinal permeability, leading to NASH.^74^ Therefore, VVYP may attenuate ethanol‐ or acetaldehyde‐induced intestinal permeability and endotoxemia through reducing the abundance of *Lactobacillus*. Pathogenic species of *Streptococcus* have been found to be related to inflammatory bowel disease, which supports a potential role of *Streptococcus* in promoting inflammation.^75^ Furthermore, LEfSe analysis indicated that treatment with VVYP had greater abundance of *S24‐7*, *Eubacteriaceae*, C*oriobacteriacease* and *Desulfovibrionaceae* at family level as well as *Bacteroidia* at class level. *Eubacteriaceae* is a critical component of a normal healthy intestine.^1^, ^2^, ^76^ Additionally, *Coriobacteriaceae*, certain species of which have been proved to be beneficial to host lipid metabolism,^78^ were increased after butyrate treatment.^25^ It is well known that another important factor contributing to changes in the liver expression of genes is the interplay between gut microbiota and bile acid (BA) metabolism, protective gut microbiota(*Desulfovibrionaceae* and *Coriobacteriaceae*) associates with increased specific secondary BAs, which likely inhibit lipogenic pathways and enhance bile flow in the liver..^79^ On the other hand, short chain fatty acids (SCFAs) derived from gut microbiota are involved in the pathogenesis of NASH, SCFA‐producing bacteria, such as *S24‐7* ^80^ and *Bacteroidaceae*,^81^ could play beneficial roles in stimulating the immune response and protecting the mucosal barrier in mice. Our results indicated that VVYP treatment could restore gut microbiota by strongly enhancing the abundance of secondary BA‐producing bacteria (*Coriobacteriacease* and *Desulfovibrionaceae*) and SCFA‐producing bacteria (*S24‐7* and *Bacteroidia*). Here, we also found VVYP enhanced the abundant of *Eubacteriaceae*, *coriobacteriacease, Desulfovibrionaceae, S24‐7* and *Bacteroidia*, the mechanism may be involved in BA/SCFA homoeostasis and ultimately exert a protective effect in NASH. The above evidence further supported that VVYP may alleviate liver injury in mice with NASH through restoring the imbalance of intestinal bacterial structure.

Previous studies demonstrated that another crucial participator in the progression of NASH is the network of pro‐inflammatory chemokines and cytokines.^82^, ^83^ Several inflammatory factors, such as TNF‐α, IL‐1β and IL‐6, have been proved to enable steatosis and liver damage, thus promoting the occurrence and progression of NASH.^84^, ^85^ In addition, TNF‐α was supposed to be a pivotal mediator of NASH development,^86^ and inhibition of TNF‐α activity by anti‐inflammatory drugs ameliorated inflammation, liver damage and NASH.^87^ Consistent with previous researches, mice fed a MCD diet effectively developed marked hepatic inflammation that simulated the natural development of NASH in human.^88^, ^89^ To investigate whether the mitigative effect of VVYP could alleviate hepatic inflammation, the TNF‐α and IL‐6 levels in liver tissue were assayed by ELISA. The results of our study indicated that VVYP treatment significantly inhibited the TNF‐α and IL‐6 levels induced by MCD diet. The gut barrier dysfunction results in a higher level of circling bacterial endotoxins, which plays an crucial role in triggering the liver inflammatory response.^90^ A recent research has indicated that MCD diet caused dysbiosis of gut microbiota and disrupted intestinal barrier function by down‐regulating expression of intestinal tight junction mRNA levels (claudin‐1 and ZO‐1) in mice.^25^ Another study has reported that the levels of claudin‐1, ZO‐1 and occludin were decreased in high‐fat and fructose diet group.^91^Once this barrier was disrupted, overproduction of LPS induced by the overgrowth of gut Gram‐negative bacterial would enter into blood and promote inflammation which impaired intestinal barrier integrity.^92^ Here, we observed an obvious reduction in mRNA levels of claudin‐1, ZO‐1 and occludin in the MCD group, while VVYP reversed these changes, and stabilized structure of tight junctions was demonstrated by organized localization and smooth of ZO‐1. The intestinal membrane‐associated Muc (Muc‐1) and the secreted Muc (Muc‐2) played a critical role in maintaining the intestinal barrier function.^23^ Our results also indicated that VVYP stimulated the mRNA expressions of Muc‐1 and Muc‐2. Many studies indicated that endotoxins could interact with TLR4, cluster of differentiation 14 (CD14) and other receptors, and ultimately promoted the activation of nuclear factor‐κB (NF‐κB) and the trigger of subsequent inflammatory gene overexpression.^93^ In the development of NAFLD, the endotoxin‐TLR4‐NF‐κB pathway is considered as the critical factor to link intestinal microbiota dysbiosis and liver inflammation.^94^ Interestingly, we also found MCD diet up‐regulated LPS levels in the liver tissues of mice in MCD group while VVYP significantly reversed these effects. Elevated liver localization of LPS was recently displayed in the patients with NAFLD and experimental NAFLD, which was connected with liver inflammation via a transport of TLR4‐mediated pathway.^95^ In our study, VVYP inhibited the increased levels of TNF‐α, IL‐6 and LPS induced by MCD diet, which was possible linked to dysbiosis‐mediated activation of the TLR4‐NF‐κB signalling pathway.

In conclusion, we have demonstrated that VVYP inhibited the cytotoxicity and lipid accumulation in L‐02 cells exposed to FFA. Noticeably, VVYP could protect against NASH modulate the gut microbiota imbalance by strongly enhancing the abundance of *Eubacteriaceae*, secondary BA‐producing bacteria (C*oriobacteriacease* and *Desulfovibrionaceae*) and SCFA‐producing bacteria (*S24‐7* and *Bacteroidia*), and VVYP could reduce the abundance of *Lactobacillus*, the mechanism may be related to modulation of ethanol‐ or acetaldehyde‐induced intestinal permeability and endotoxemia. These results confirmed the important role of intestinal flora in regulating the occurrence and progression of NASH. Moreover, VVYP mitigated the damage of intestinal barrier as well as inhibited the subsequent expression of inflammatory cytokines in the liver tissues of mice fed by MCD diet. Our study highlights the protective role of VVYP against NASH in vitro and in vivo, which may provide new strategy for preventing NASH development and progression based on the gut‐liver axis.

## CONFLICT OF INTEREST

The authors declare no conflicts of interest.

## AUTHOR CONTRIBUTIONS

**Xinshu Xie:** Writing‐original draft (equal). **Lang Zhang:** Visualization (equal). **Shun Yuan:** Validation (equal). **Huilan Li:** Validation (equal). **Chaojun Zheng:** Validation (equal). **Saisai Xie:** Writing‐review & editing (equal). **Yongbing Sun:** Validation (equal). **Changhua Zhang:** Software (equal). **Rikang Wang:** Writing‐review & editing (equal). **Yi Jin:** Supervision (equal).

## Data Availability

All data generated or analysed during this study are in this paper.
